# Importin α3 (KPNA3) Deficiency Augments Effortful Reward-Seeking Behavior in Mice

**DOI:** 10.3389/fnins.2022.905991

**Published:** 2022-06-30

**Authors:** Yoshiatsu Aomine, Koki Sakurai, Tom Macpherson, Takaaki Ozawa, Yoichi Miyamoto, Yoshihiro Yoneda, Masahiro Oka, Takatoshi Hikida

**Affiliations:** ^1^Laboratory for Advanced Brain Functions, Institute for Protein Research, Osaka University, Osaka, Japan; ^2^Department of Biological Sciences, Graduate School of Science, Osaka University, Osaka, Japan; ^3^Laboratory of Nuclear Transport Dynamics, National Institutes of Biomedical Innovation, Health and Nutrition (NIBIOHN), Osaka, Japan; ^4^National Institutes for Biomedical Innovation, Health and Nutrition (NIBIOHN), Osaka, Japan

**Keywords:** importin α, KPNA, progressive ratio schedule, c-fos, functional connectivity, brain network, centrality

## Abstract

Importin α3 (Gene: *Kpna3*, the ortholog of human Importin α4) is a member of the importin α family and participates in nucleocytoplasmic transport by forming trimeric complexes between cargo proteins and importin β1. Evidence from human studies has indicated that single nucleotide polymorphisms (SNP) in the *KPNA3* gene are associated with the occurrence of several psychiatric disorders accompanied by abnormal reward-related behavior, including schizophrenia, major depression, and substance addiction. However, the precise roles of importin α3 in controlling reward processing and motivation are still unclear. In this study, we evaluated the behavioral effects of *Kpna3* knockout (KO) in mice on performance in touchscreen operant chamber-based tasks evaluating simple (fixed-ratio) and effortful (progressive-ratio) reward-seeking behaviors. While *Kpna3* KO mice showed no significant differences in operant reward learning on a fixed-ratio schedule, they demonstrated significantly increased motivation (increased break point) to instrumentally respond for sucrose on a progressive-ratio schedule. We additionally measured the number of c-Fos-positive cells, a marker of neural activity, in 20 regions of the brain and identified a network of brain regions based on their interregional correlation coefficients. Network and graph-theoretic analyses suggested that *Kpna3* deficiency enhanced overall interregional functional connectivity. These findings suggest the importance of *Kpna3* in motivational control and indicate that *Kpna3* KO mice may be an attractive line for modeling motivational abnormalities associated with several psychiatric disorders.

## Introduction

Importin α3 (Gene Symbol: *Kpna3*; the ortholog of human importin α4) is a member of the importin α family of nuclear transport factors (Miyamoto et al., 2016). Importin α family proteins recognize and bind classical nuclear transport signals (cNLS) of nucleoproteins and act as adaptor molecules to importin β (Goldfarb et al., 2004). The formation of cargo-importin α-importin β trimeric complexes allows cargo proteins to enter the nucleus by passing through Nuclear Pore Complexes (NPCs) found on the nuclear membrane (D’Angelo and Hetzer, 2008; Raices and D’Angelo, 2012; Christie et al., 2016). Importin α subtypes (6 in mice and 7 in humans) act to determine cellular function through transport regulation of transcription factors (Yasuhara et al., 2007, 2013), and have been reported to demonstrate partially redundant, yet differential, cellular specificity (Pumroy and Cingolani, 2015).

In mice, 3 of the 6 identified importin α subtypes show higher expression than other subtypes in the brain (importin α1/KPNA1, importin α3/KPNA3, importin α4/KPNA4) (Hosokawa et al., 2008; Moriyama et al., 2011; Thiele et al., 2020). Although the neural functions of importin αs in the brain are yet to be fully understood, recent studies using knockout mouse lines have revealed these proteins to play important roles in controlling emotional behavior and nociception. Panayotis et al. (2018) and Sakurai et al. (2021) revealed importin α1 (*Kpna1*) deficiency to cause decreased anxiety-related behavior, which in Panayotis et al. (2018), was associated with hippocampal presynaptic hypofunction, impaired long-term potentiation, and decreased nuclear transportation of a specific transcription factor [Methyl CpG binding protein 2 (MeCP2)]. In another study, Marvaldi et al. (2020) revealed that importin α4 (*Kpna4*)-deficient mice show decreased pain sensitivity in dorsal root ganglion neurons. Despite its ubiquitous expression throughout the brain (with the exception of the olfactory bulb), the role of importin α3 (*Kpna3*) in behavioral regulation has not yet been characterized.

Interestingly, independent studies have identified single nucleotide polymorphisms (SNPs) of *KPNA3*, predicted to decrease KPNA3 expression, to be associated with schizophrenia in cohorts from the United Kingdom, China, and Australia (Wei and Hemmings, 2005; Zhang et al., 2006; Morris et al., 2012). Furthermore, in the Australian cohort, *KPNA3* SNPs were additionally associated with a wide range of other psychiatric disorders, including alcoholism, opioid addiction, and major depression (Morris et al., 2012). Such insights suggest *KPNA3* to be a possible genetic risk factor for psychiatric disorders; however, the causal relationship between KPNA3 and such disorders is still unknown.

Recent considerations on the psychological mechanisms underlying major psychiatric disorders such as schizophrenia, depression, bipolar disorder, and substance addiction have suggested impaired reward processing as a shared malfunction of the brain across multiple psychiatric disorders (Gardner, 2011; Volkow and Morales, 2015; Whitton et al., 2015; Lambert et al., 2018; Robison et al., 2020). As all 4 disorders significantly associated with *KPNA3* involve malfunctions in reward processing, *KPNA3* depletion may lead to altered reward-related behavior.

In this study, we examined reward-related behavior in *Kpna3* KO mice to assess how *Kpna3* deficiency alters the brain’s reward system. Assessment of reward-related motivation using fixed and progressive ratio schedule operant tasks revealed augmented instrumental responding for rewards in the progressive ratio task in *Kpna3* KO mice. As a wide range of brain regions are associated with reward-related behavior, we then quantified c-Fos-positive cells to assess neural activity during the progressive ratio task in 20 distinct brain regions, including regions involved in the control of reward-related behavior (such as emotion and cognition) and feeding. From this c-Fos mapping data, we constructed a functional connectivity network based on interregional correlations of activity (number of c-Fos-positive cells). These data provide novel insight into the role of *Kpna3* in reward-related motivation, and reveal that *Kpna3* deficiency, such as that which may occur as a result of *Kpna3* SNPs associated with psychiatric disorders, causes brain network alterations that co-occur with augmented motivation.

## Materials and Methods

### Animals

Homozygous *Kpna3* knockout (KO) and wild type (WT) mice on a C57BL6/NJcl (CLEA Japan Inc., Tokyo, Japan) background were generated at the Institute for Protein Research, Osaka University, by crossing male and female *Kpna3* heterozygous mice. The generation of *Kpna3* KO mice (12B1 line) and genotyping methods have been previously described (Miyamoto et al., 2020). Mice were housed on a 12-h light/dark cycle (Light: 0800–2000, Dark: 2000–0800) in a quiet environment with room temperature maintained at 24°C ± 2°C. All mice were weaned and housed with their same-sex littermates with *ad libitum* access to food and water until the start of behavioral experiments. Experiments were performed on male mice after reaching 8 weeks of age. All animal experiments complied with institutional guidelines set by Osaka University Living Modified Organisms (LMO) Research Safety Committee and Osaka University Institute for Protein Research Animal committee.

### Pre-training Fixed Ratio Schedule

Mice (WT = 10, KO = 9) were individually housed at least 3 days prior to the start of the experiment. Food consumption was restricted to maintain mice at 80–90% of their initial free-feeding body weight. Mice were fed every day after the completion of the task. Food was given immediately after the end of the task, i.e., all mice were removed from the chamber and had access to food as soon as they returned to their home cages. Water was always available in the home cage. The progressive ratio is exponential. Experiments were conducted in mouse touch screen chambers (Model 80614, Campden Instruments Ltd., England). A partition plate with two square holes (W:7 cm, H:7.5 cm) in the center with a 5 mm space in between was fitted in front of the touch screen to create two distinct panels for touch response (left/right). A dish for reward presentation was placed on the opposite side of the touch screen. The chamber was always kept dark during experiments, with only two light sources: a touch screen light (panel lights) and a light on the top of the reward presentation dish (dish light).

In the FR-1, FR-2, and FR-3 schedules, mice received a reward (20 μl of 10% sucrose solution) after touching the panel paired with the reward (correct panel) 1, 2, or 3 times, respectively. The other panel (incorrect panel) was not paired with any reward/punishment. The side of the correct panel (left/right) was counterbalanced among mice to offset any individual side preferences. Trials were initiated with the illumination of panel lights. After touching the correct panel n times, the panel lights were switched off, and the reward was presented together with illumination of the dish light. The dish light was switched off after the reward was collected to finish the trial and mice underwent a 30-s inter-trial interval before the start of the next trial. On the first day, mice underwent 15 h of training on an FR-1 schedule. Subsequently, training was conducted once daily beginning at an FR-1 schedule, and then progressing to FR-2 and FR-3 schedules. Each session lasted 60 min or until 100 reward collections had been achieved. Mice proceeded to the next stage when they reached 50 reinforcements (FR-1,2) or 50 reinforcements for 2 consecutive days (FR-3).

### Progressive Ratio Schedule

The progressive ratio (PR) operant task was started after the successful completion of an FR-3 schedule. In the PR schedule, the number of panel touches required to earn rewards (response ratio, P_m_) was determined according to the following formula (e.g., mice are presented with a reward after a single touch in the first trial, but the number of touches required for consecutive reward presentation increases gradually 1, 2, 4, 6, 9, 12, 15, 20, …) (Richardson and Roberts, 1996). m is the order number of a trial.

$$P_{m}=\left[5\,e^{\left(m\times 0.2\right)}\right]-5$$

The task was terminated when mice failed to touch either panel for over 5 min, or after a duration of 60 min, whichever came first. The response ratio for the last reward collection was designated as the break point (limit of the effort the animal will expend to gain the reward). When the number of rewards earned in a session did not change by more than 10% for 3 consecutive days, the performance on the PR schedule was considered to be stable and used for analysis. All PR data were averaged over 3 days.

### Immunohistochemistry

Ninety minutes after the completion of the last PR schedule session, mice were anesthetized with isoflurane and transcardially perfused with phosphate-buffered saline (PBS) then PBS containing 4% paraformaldehyde (PFA) (Nacalai Tesque, Inc., Kyoto, Japan). Brains were removed and immersed in PBS containing 4% PFA overnight at 4°C for post-fixation then kept in PBS until use. For cryosectioning, brains were immersed in 30% sucrose containing PBS for 24 < h, flash-frozen in –80°C isopentane, embedded in embedding agent (Tissue-Tek O.C.T compound, Sakura Finetek Japan Co., Ltd., Tokyo, Japan), and sliced into 30 μm thick sections on a cryostat microtome (Leica CM1860, Leica Biosystems). For staining, sections were washed three times for 5 min in PBS, followed by blocking and antigen activation in PBS containing 10% normal goat serum (NGS) + 0.1% Triton-X100. Anti-c-Fos primary antibody [c-Fos(9F6) Rabbit mAb #2250, 1/1,000, Cell Signaling Technology] and NeuN antibody (MAB377 Anti-NeuN Antibody, clone A60, 1/500, Sigma-Aldrich, Merck) was then added and sections were incubated at 4°C for 48 h. Sections were washed three times with PBS for 5 min and incubated in PBS containing secondary antibody (Goat anti-Rabbit IgG(H + L) Alexa Fluor 488, 1/500, Invitrogen, Thermo Fisher Scientific) + 0.5% NGS + 0.1% Triton-X100 for 2 h at room temperature. Finally, sections were washed 3 times for 5 min with PBS, mounted onto glass slides, and coverslipped with glass covers and DAPI-containing mounting medium (ab104139, Abcam). Reagent solutions used for staining were prepared in large quantities at a time, and the same solution was used to stain WT and KO samples simultaneously to minimize variation.

### c-Fos Counting

Immunostained sections were photographed on a fluorescence microscope (BZ-X810, KEYENCE CORPORATION, Osaka, Japan) using a 20x objective lens. Z-stacked images from neighboring areas were stitched together using software (Image Analyzer ver1.1.1, KEYENCE CORPORATION, Osaka, Japan) to create high resolution images for each region. A total of six images were acquired bilaterally for each region (three images for dorsal and median raphe nucleus), and the number of c-Fos positive cells per region was counted manually. The names and labels of image files were randomized so that the experimenter analyzing the c-Fos positive cells was unaware of the genotype of the mouse. The average number of c-Fos-positive cells across all images was calculated for each area. Counts were performed in the following 20 regions, VO/LO: ventral and lateral orbital area, MO: medial orbital area, NAc (a-m, a-l, p-m, p-l, and p-v): nucleus accumbens, VP: ventral pallidum, MPOA: medial preoptic area, dHip: dorsal hippocampus, vHip: ventral hippocampus, BLA (a and p): basolateral amygdala, LH (a and p): lateral hypothalamus, VMH: ventromedial hypothalamic nucleus, ARC: arcuate nucleus, VTA: ventral tegmental area, DR: dorsal raphe nucleus, MR: median raphe nucleus (a: anterior, p: posterior, m: medial, l: lateral, v: ventral).

### Statistical Analysis

GraphPad Prism software (v. 8.0.1, GraphPad Software Inc.) was used for all statistical analyses. When genotype was the only grouping variable, an independent two-tailed Student’s *t*-test was used. Survival curves were tested using the log-rank test. Two-way ANOVA and Bonferroni tests for multiple comparisons were used for the analysis of c-Fos-positive cells. As the correlation coefficients of one region with all other regions were considered not to follow a normal distribution, Mann-Whitney U-tests were used to compare entire correlation coefficients. And Mann-Whitney U-tests with Bonferroni correction for multiple comparisons were used to compare correlation coefficients in each region.

### Comparison of Correlation Matrices

Pearson product-moment correlation coefficients (r) were calculated for all combinations of regions and a correlation matrix was generated for each of the WT and KO groups. We used multiple individuals to determine the correlation between regions in the brains of either WT and KO to perform intra-individual analysis within WT and KO individuals (Supplementary Figure 6).

$$\text{r}=\frac{S_{x\,y}}{S_{x}\,S_{y}}=\frac{\frac{1}{n}\,\sum_{i=1}^{n}\left(x_{i}-x\right)\,\left(y_{i}-y\right)}{\sqrt{\frac{1}{n}\,\sum_{i=1}^{n}{\left(x_{i}-x\right)}^{2}}\,\sqrt{\frac{1}{n}\,\sum_{i=1}^{n}{\left(y_{i}-y\right)}^{2}}}$$

x and y are the numbers of c-fos positive cells in each region, S_xy_ is the covariance of x and y, S_x_ is the standard deviation of x, S_y_ is the standard deviation of y, n is the total number of bivariate data (x, y) and is 20 for the WT group and 18 for the KO group, x_i_ and y_i_ are the individual values, and x and y are their respective means. The correlation matrices were used to compare the correlation of each region with other regions. Genotype-dependent changes were assessed by comparing Pearson product-moment correlation coefficients (r) between genotypes.

The correlation coefficient is a unitless measure of the relationship between two types of data indicating the relationship between the data without being affected by unit. Therefore, the correlation coefficient does not depend on the variance of the data (variation in the number of neurons) or the overall height of the data (number of neurons).

### Network Construction and Graph Theoretical Analysis

The network analysis was carried out according to previously reported methods with minor modifications (Tanimizu et al., 2017; Kimbrough et al., 2020). Correlations between regions were filtered at an uncorrected significance level of *p* < 0.05. Details of the test are given below.

null hypothesis: *r* = 0 as the mother correlation coefficient is 0 (no correlation exists). Alternative Hypothesis: *r* ≠ 0 as the mother correlation coefficient is not 0 (correlation exists). The t-statistic is calculated by the following equation. *p*-value is the probability that a value greater than or equal to |t| occurs in the *t*-distribution. *r*: correlation coefficient, *n*: number of individuals.

$$\text{t}=\frac{r\,\sqrt{n-2}}{\sqrt{1-r^{2}}}$$

Networks were constructed for each of the WT and KO groups using the correlation coefficient of the number of c-Fos-positive cells between significantly correlated regions as edges (edges, branches, lines) and each region as a node (vertices, nodes). Using this network, two types of centralities were analyzed by graph theoretic analysis. Degree centrality is the number of edges connected to each node (number of edges = degree centrality), representing its direct influence on its neighbors, whereas betweenness centrality is a measure of the proportion of a node included in the shortest path between other nodes. It detects nodes that are important for the indirect connection of other nodes, such as those belonging to several modules (Joyce et al., 2010). These two centrality measures were used to assess the influence of each area within each network. Graph theoretical analysis and visualization of the graphs were performed using Cytoscape (version 3.9.0).

### Reproducibility Evaluation of the Centrality Score With the Bootstrap Method

To confirm the reproducibility of the results in the network analysis, an evaluation using the bootstrap method was performed. For each WT and KO sample, 380 bootstrap samples were resampled. During the resampling process, one area was duplicated and one area was deleted. Networks were constructed for all samples and order centrality and mediation centrality were determined based on resampled data. Rstudio (R, version 3.6.0) and igraph (version 1.3.1) were used for analysis.

## Results

### *Kpna3* Knockout Mice Show Normal Operant Learning on a Fixed Ratio Schedule

**FIGURE 1:**
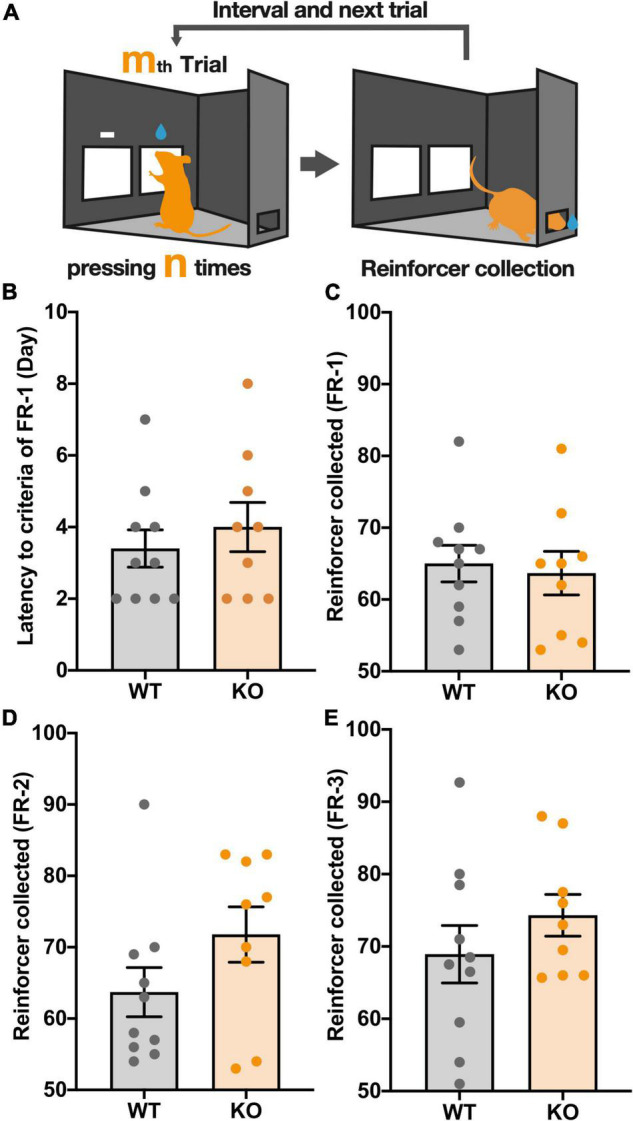
KPNA3 KO mice show unaltered performance in a fixed ratio (FR) schedule test when compared with WT mice. **(A)** Illustration of The FR schedule test. The right panel is the correct panel and the left is the incorrect panel (left image). n is a fixed value, 1, 2, or 3. m is the order number of a trial. Following a correct response mice can collect a reward from the rear reward magazine (right image). **(B)** The Number of days until the FR-1 criterion was reached. **(C–E)** The number of rewards earned in sessions where the criterion was reached. The criterion for FR-1 and 2 was 50 reinforcers and FR-3 was 50 reinforcers on 2 consecutive days. Data represent the mean ± SEM, Student’s *t*-test (WT = 10, KO = 9).

First, mice were tested on a fixed-ratio schedule (FR), in which a fixed number of touches (1, 2, 3 for FR-1, FR-2, FR-3, respectively) to the reward panel (response) triggered the delivery of a 10% sucrose solution (reward). Mice were considered to have successfully responded to the task if they reached a criterion of 50 reward collections per trial (Figure 1A). In FR-1, *Kpna3* KO mice did not differ significantly from controls (WT) in the number of rewards earned and the number of days taken to reach the criterion (Figures 1B,C, number of rewards earned: *t*_17_ = 0.3387, *p* = 0.739; number of days to reach criterion: *t*_17_ = 0.7046, *p* = 0. 4906). Mice were then underwent FR-2 and FR-3 schedules, where again no significant difference in the number of rewards earned was observed for both FR-2 and FR-3 (Figures 1D,E, FR-2: *t*_17_ = 1.561, *p* = 0.1369, FR-3: *t*_17_ = 1.073, *p* = 0.2981). Almost all mice subjected to the FR schedule reached the criterion in 1 day (FR-2) or 2 days (FR-3). Overall, there was no significant difference in the learning speed and ability to acquire the reward between the two groups (genotypes). Furthermore, there was no difference observed in body weight between WT and KO mice both with and without food restriction (Supplementary Figure 1).

### *Kpna3* Knockout Mice Show Higher Motivation to Acquire the Reward on a Progressive Ratio Schedule

**FIGURE 2:**
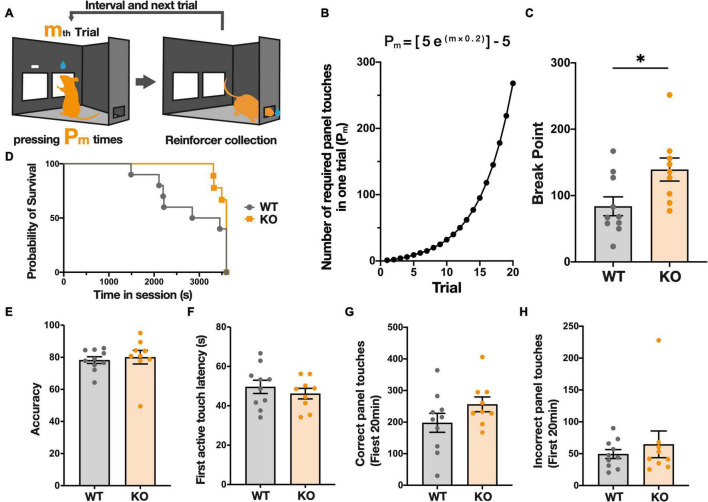
KPNA3 KO mice show increased motivation to acquire a sucrose reward in a progressive ratio (PR) schedule test in comparison to WT mice. **(A)** Illustration of The PR schedule test. The right panel is the correct panel and the left is the incorrect panel (left image). m is the order number of a trial and P_m_ is the response ratio. Following a correct response mice can collect a reward from the rear reward magazine (right image). **(B)** The ratio schedule of the PR schedule test. **(C)** Break Point: number of panel touches required to obtain the reward at the time when the mouse finishes the task. **(D)** Survival curve of the duration until the end of the session (no touch for 5 min, or 60 min). **(E)** Correct panel ratio in the entire session (correct touches/total panel touches). **(F)** Average time between the start of the trial and the first panel touch. **(G,H)** The number of correct/Incorrect touches in the first 20 min (the shortest session). Data represent the mean ± SEM, *p < 0.05, Student’s *t*-test for column data and A log-rank (Mantel-Cox) test for survival data (WT = 10, KO = 9).

After the completion of FR-3, mice were started on a PR schedule test, in which the number of operant responses to obtain a reward (reinforcer) increases with each reward collected, to evaluate the motivation of mice to instrumentally respond for a reward (Figures 2A,B). In this schedule, the break point, defined as the number of responses needed to receive the last reward collected, allows assessment of how much effort (responses) mice are willing to expend for a single reward. In the PR schedule, *Kpna3* KO mice showed a higher break point than WT mice (Figures 2C,B; KO vs. WT, *t*_17_ = 2.494, *p* = 0.0232). The duration of the trial did not differ significantly, as shown in the survival curves in Figure 2D (chi-square χ2 = 2.112, *p* = 0.1461, maximum trial duration 60 min). Analysis using the ROUT test for outlier removal (*Q* = 1%) resulted in no data points being excluded as outliers, suggesting that the increase in breakpoint in KO mice is not due to the effect from outliers (Supplementary Figure 2). There was no significant difference between KO and WT mice in all other measures, including accuracy (total number of reward panel/panel contacts) (Figure 2E, *t*_17_ = 0.3937, *p* = 0.6987), active touch latency (time between the trial initiation and first panel touch) (Figure 2F, *t*_17_ = 0.7873, *p* = 0.442), number of correct panel contacts in the first 20 min (Figure 2G, *t*_17_ = 1.511, *p* = 0.1492), and the number of contacts with incorrect panel in the first 20 min (Figure 2H, *t*_17_ = 0.7205, *p* = 0.481). These findings indicate that *Kpna3* KO mice showed augmented motivation to instrumentally respond for the reward, but no change in the ability to correctly discriminate between the two panels or in reaction times.

### No Significant Change in the Number of Activated Cells During Progressive Ratio Schedule in *Kpna3* Knockout Mice

**FIGURE 3:**
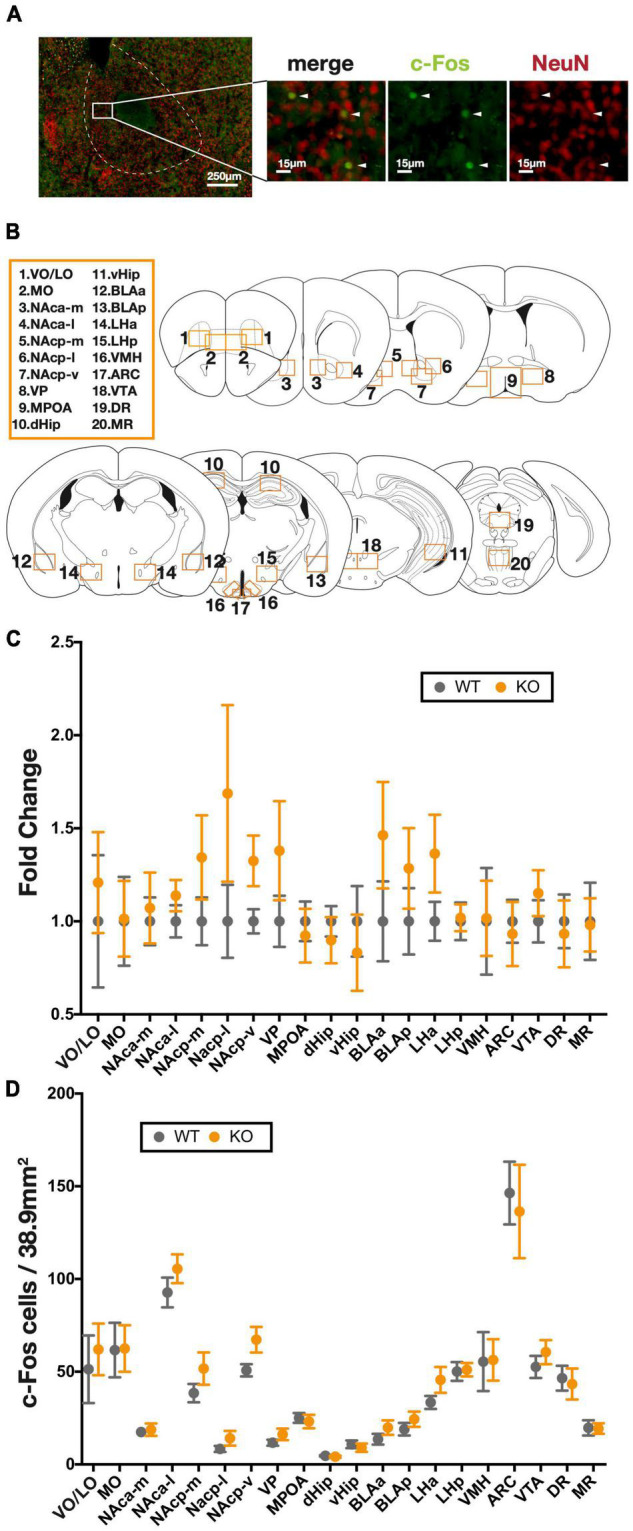
c-Fos mapping of 20 brain regions following the progressive ratio schedule test. **(A)** Representative image of c-Fos positive cells, white head arrows indicate counted cells. **(B)** Twenty brain regions in which c-Fos-positive cells were counted. **(C)** Fold change from WTs in the number of c-Fos-positive cells in each region. **(D)** The number of c-Fos positive cells in each region. Data represent the mean ± SEM, two-way ANOVA and genotype effect at each region, a *post hoc* Bonferroni multiple comparison test (WT = 10, KO = 9). ARC, arcuate nucleus; BLA, basolateral amygdala; dHip, dorsal hippocampus; DR, dorsal raphe nucleus; LH, lateral hypothalamus; MO, medial orbital area; MPOA, medial preoptic area; MR, median raphe nucleus; NAc, nucleus accumbens (a, anterior; *p*, posterior; m, medial; l, lateral; v, ventral), vHip, ventral hippocampus; VMH, ventromedial hypothalamic nucleus; VO/LO, ventral and lateral orbital area; VP, ventral pallidum; VTA, ventral tegmental area.

The expression of the immediate early gene, c-Fos, is a well-known marker of neural activity (Morgan and Currant, 1986; Morgan et al., 1987). In order to assess neural activity during the PR task, we performed anti-c-Fos immunostaining on brain sections collected 90 min after the completion of the final PR task (Figure 3A). As recent studies have highlighted a wide range of brain regions related to reward-related behavior (Camara et al., 2009), we performed c-Fos counting in a total of 20 regions (Figure 3B) associated with reward pathways to determine possible brain regions implicated with PR performance, as well as their possible alteration as a result of *Kpna3* depletion. In a two-way RM ANOVA analysis, there was found to be no significant main effects of genotype [relative ratio: *F*(1, 17) = 1.007, *p* = 0.3298, number of positive cells: *F*(1, 17) = 0.5344, *p* = 0.4747], genotype × brain region effect [relative ratio: *F*(19, 322) = 0.9088, (19, 322) = 0.9088, *p* = 0.5722, number of positive cells: *F*(19, 322) = 0.3662, *p* = 0.9939] (Figures 3C,D). Bonferroni tests for multiple comparisons to analyze the effect of *Kpna3* depletion in each region also showed that there were no brain regions with significant differences between KO vs. WT in the number of c-Fos-positive cells.

### *Kpna3* Deficiency Increases Connectivity Between Regions During Progressive Ratio Schedule Testing

**FIGURE 4:**
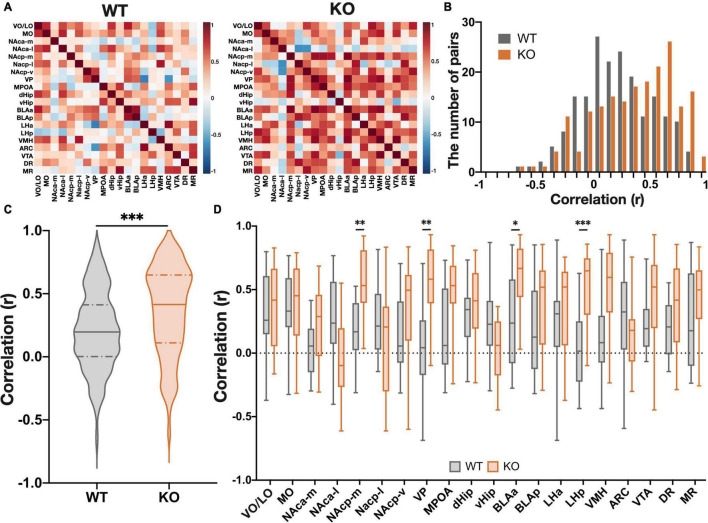
KPNA3 KO mice show increased functional connectivity during the performance in the progressive ratio schedule test when compared to WT mice. **(A)** Heat map of all interregional correlation coefficients in WT and KO groups. **(B)** Histogram of all interregional correlation coefficients. **(C)** Violin plot of all correlation coefficients. Solid line: median, dashed line: quartiles. Mann Whitney u test. **(D)** Correlation coefficients of each region. Data represent the median, quartiles, maximum, and minimum, Mann Whitney u test and Bonferroni correction, *p < 0.05, **p < 0.01, ***p < 0.001. ARC, arcuate nucleus; BLA, basolateral amygdala; dHip, dorsal hippocampus; DR, dorsal raphe nucleus; LH, lateral hypothalamus; MO, medial orbital area; MPOA, medial preoptic area; MR, median raphe nucleus; NAc, nucleus accumbens (a, anterior; *p*, posterior; m, medial; l, lateral; v, ventral), vHip, ventral hippocampus; VMH, ventromedial hypothalamic nucleus; VO/LO, ventral and lateral orbital area; VP, ventral pallidum; VTA, ventral tegmental area.

Reward-seeking behavior and its motivation are regulated not only by single brain regions, but also by activity networks comprising of multiple brain regions (Camara et al., 2009). Therefore, in order to identify brain-wide changes in neural activity that underlie disorders of reward-related behavior, it is necessary to investigate the activity of inter-regional networks. Graph-theoretic centrality of the c-Fos network has been utilized to identify regions that produce specific behavioral changes in mice (Tanimizu et al., 2017; Kimbrough et al., 2020), and previous research has demonstrated that centrality in the c-Fos-based inter-regional network correlates with the amount of influence on a specific behavior when the region is chemogenetically silenced during behavior (Vetere et al., 2017). This implies that hub regions with high centrality in a network based on measures of functional connectivity play a central role (influence) in controlling specific behaviors. Functional connections consist of either direct neural connections and indirect connections between brain regions, with regions with more functional connections to other regions primarily controlling specific behaviors. To estimate functional connectivity between brain regions during the PR schedule test, we calculated the correlation coefficients of the number of c-Fos positive cells between all measured brain regions in both WT and KO mice (Figure 4A). A comparison between groups (genotype) of the total number of inter-regional correlations showed a greater number of positive correlations in *Kpna3* KO mice (Figures 4B,C, *p* < 0.0001). We further analyzed the effects of genotype on the correlation coefficients in each region relative to other regions. Significant increases in correlation coefficients with other regions were found in the medial nucleus accumbens (NAcp-m: *p* = 0.0028), ventral pallidum (VP: *p* = 0.0020), amygdala (BLAa: *p* = 0.026) and lateral hypothalamus (LH-p: *p* = 0.0006). This suggests that *Kpna3* deficiency strengthens the connections of these regions to other regions during PR schedule testing (Figure 4D).

**FIGURE 5:**
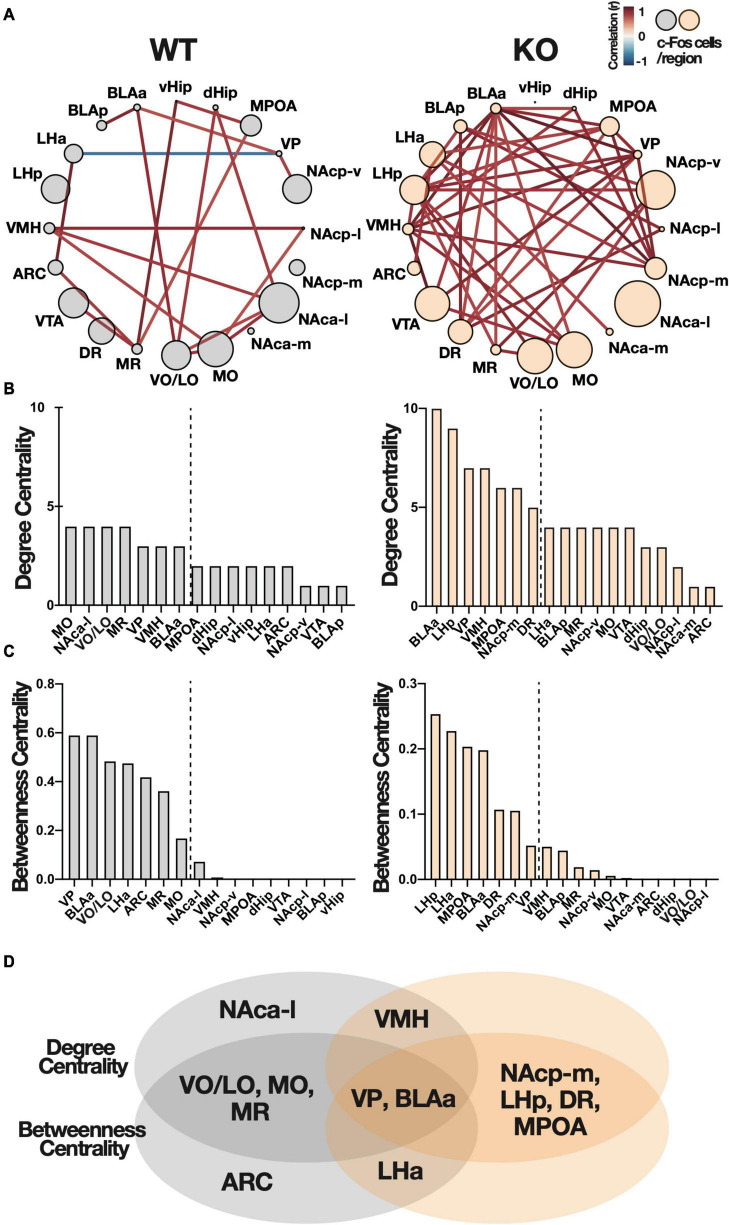
Functional connectivity networks in the brain during the progressive ratio schedule test. **(A)** Networks with brain regions as nodes and inter-regional connections as edges (significant correlation coefficient: *p* < 0.05). The color of the edges represents the correlation coefficient and is indicated by the heat map in the top right (Figure 2A). The size of the node indicates the average number of c-Fos positive cells in each region. **(B,C)** Degree centrality and betweenness centrality of each region in the network. The broken line is the boundary of the seven regions of high centrality. **(D)** The Venn diagram shows the overlap in the seven regions of high centrality. ARC, arcuate nucleus; BLA, basolateral amygdala; dHip, dorsal hippocampus; DR, dorsal raphe nucleus; LH, lateral hypothalamus; MO, medial orbital area; MPOA, medial preoptic area; MR, median raphe nucleus; NAc, nucleus accumbens (a, anterior; *p*, posterior; m, medial; l, lateral; v, ventral), vHip, ventral hippocampus; VMH, ventromedial hypothalamic nucleus; VO/LO, ventral and lateral orbital area; VP, ventral pallidum; VTA, ventral tegmental area.

Based on these c-Fos correlation matrices, we generated networks with brain regions as nodes and inter-regional connections (significant correlation coefficient: *p* < 0.05) as edges. An increase in network density was observed in *Kpna3* KO mice compared to WT mice (Figure 5A, number of edges: WT = 19, KO = 37). This network was used for graph-theoretic analysis (Figures 5B,C). Degree centrality is defined as the number of edges connected to each node (number of edges = degree centrality), in other words, an increase in edges is synonymous with an increase in degree centrality, and betweenness centrality is defined as the proportion of one node included in the shortest path between other nodes. It should be noted that degree centrality was comparable between WT and KO, whereas betweenness centrality differed due to the number of edges. We defined high centrality as being ranked within the top 35% on both centralities (Figures 5B–D). The ventral pallidum (VP) and the amygdala (BLAa) show high centrality in both WT and KO groups. Whereas the medial posterior nucleus accumbens (NAcp-m), medial preoptic area (MPOA), posterior lateral hypothalamus (LHp), and dorsal raphe nucleus (DR) show high centrality only in the KO group. Finally, the ventral and lateral orbital area (VO/LO), medial orbital area (MO), and median raphe nucleus (MR) show high centrality only in the WT group. These results indicate that *Kpna3* deficiency increases the overall functional connectivity between regions as well as the centrality within the network in specific regions during PR schedule testing. Evaluation of the reproducibility of these data by the bootstrap method showed no change in the order of centrality (Supplementary Figure 3).

## Discussion

Here, for the first time, we examined potential abnormalities in reward-related behavior as a result of KPNA3 deficiency, which had previously been genetically implicated in the etiology of several psychiatric disorders in humans. Effortful reward-seeking behavior was investigated in a touchscreen progressive ratio schedule in *Kpna3* KO mice, and task-related neural activities were measured based on c-Fos protein expression. We revealed *Kpna3* KO mice to demonstrate a higher break point than WT mice, suggesting *Kpna3* deficiency increases the motivation to instrumentally respond for a sucrose reward. This is the first report to use an animal model of *KPNA3* deficiency and supports the statistical evidence reported in human genetic studies that *KPNA3* is associated with reward-related behaviors and their dysfunction (Wei and Hemmings, 2005; Zhang et al., 2006; Morris et al., 2012). We also found that correlation coefficients of neural activity among all regions were generally increased in KO mice, and that the density of functional connectivity networks was also increased. These findings suggest that *Kpna3* deficiency enhances the overall connectivity between brain regions implicated in motivational control. Furthermore, subsequent network analysis identified hub brain regions, such as the posterior medial nucleus accumbens, to underly augmented motivation in KO mice.

A heightened break point on a progressive ratio schedule in KO mice could also be explained by changes in cognitive or hyperactive factors. Therefore, to investigate this possibility, we analyzed several other behavioral measurements (i.e., latency to criteria, reinforcer collected, accuracy, first active touch latency) in the fixed ratio schedules and a subsequent progressive ratio schedule. Our findings indicated that the basic appetitive behaviors such as operant learning, ability to earn rewards, and hyperactive behavior were unaffected by *Kpna3* deletion. In summary, we found *Kpna3* deficiency to specifically alter motivational vigor in a progressive ratio schedule, increasing the amount of effort that mice would expend to acquire a sucrose reward. In addition, given that heightened reward seeking in *Kpna3* KO mice was observed only when the cost of obtaining reward is high in the PR test, it is also possible that *Kpna3* KO mice has altered cost/benefit decision-making process such as fewer sensitivities to the behavioral cost and to the omission of expected reward. Therefore, KPNA3 might function to regulate adaptive motivational and/or decision-making processes.

To investigate the neural mechanisms underlying this increased motivation in the PR test in KO mice, we analyzed the number of neurons activated during a progressive ratio schedule in 20 brain regions. Our analysis found no significant difference between KO and WT mice in the number of c-Fos-expressing cells in any of the brain regions, suggesting that elevated motivation in KO mice cannot merely be explained by an increase of neural activity in a specific brain region. Next, we compared correlation coefficients among all brain regions and found an overall increase in correlation coefficients in KO mice. In particular, correlations were significantly increased in four regions: the posterior medial nucleus accumbens (NAcp-m), ventral pallidum (VP), anterior basolateral amygdala (BLAa), and posterior lateral hypothalamus (LHp). This indicates that the neural activities of these brain regions were more efficiently linked to the activities of other brain regions in *Kpna3* KO mice.

We then constructed a functional connectivity network to better understand changes in functional connectivity between brain regions as a result of *Kpna3* deletion. Previous studies have shown that motivation, including effort-based reward-seeking behavior, is controlled by complex neural circuits consisting of multiple regions, including the basal ganglia, cortex, amygdala, hypothalamus, and hippocampus (Macpherson and Hikida, 2019; Simmler and Ozawa, 2019; Macpherson et al., 2021). In complex neural networks, brain regions communicate with other regions not only neural connections, but also indirect connections (functional connections). This means that regions with high functional connectivity contribute significantly (centrality) to the function of the neural circuit. Thus, investigating functional connectivity between brain regions in a network can potentially elucidate how different brain regions collaboratively control a specific function (Rykhlevskaia et al., 2008). This approach was used to detect hub regions by defining “degree” and “betweenness” centrality based on graph theory. It has been previously reported that highly centralized hub regions in c-Fos-based networks causally play an important role in controlling specific behaviors (Tanimizu et al., 2017; Vetere et al., 2017). In the present study, we identified three types of hub brain regions associated with effortful reward-seeking behavior (Figure 5D). In the first type, the ventral pallidum (VP) and anterior basolateral amygdala (BLAa) showed high centralities in both WT and KO mice suggesting that these two regions play a central role in the regulation of motivation in both WT and KO mice. Importantly, the degree centrality in these two regions in the KO group was more than twice of that in the WT group. Therefore, control of network activity through VP and BLAa is stronger in *Kpna3* KO mice. In the second type, the medial posterior nucleus accumbens (NAcp-m), posterior lateral hypothalamus (LH-p), medial preoptic area (MPOA), and dorsal raphe nucleus (DR) showed high centrality only in KO mice, suggesting that these regions play a central role in the control of motivation following the deletion of *Kpna3*. Finally, in the third type, the lateral and ventral orbital area (LO/VO), medial orbital area (MO), and median raphe nucleus (MR) showed high centrality only in WT mice. Importantly, previous studies have indicated that neural circuits incorporating the NAc, VP, BLA, LH, and DR play a regulatory role in reward-related behaviors. The NAc is known to integrate information from multiple cortical and limbic brain regions and transmits information *via* direct and indirect output pathways to the substantia nigra and ventral tegmentum, where dopaminergic nuclei reside (Macpherson et al., 2014; Nakanishi et al., 2014; Salamone et al., 2018). It has been reported that overexpression of cAMP response element-binding protein (CREB) and optogenetic activation of dopamine D1 receptor-expressing neurons (contained in the direct pathway) in the NAc increase the break point during a PR task (Larson et al., 2011; Soares-Cunha et al., 2016). The VP is a downstream target of the NAc and DREADD inhibition, optogenetic activation during reward prediction cues, and optogenetic inhibition of activity during reward consumption, in D2-neurons projecting from the NAc to the VP increases the break point in a PR task (Gallo et al., 2018; Soares-Cunha et al., 2022). Furthermore, inhibition of GABA neurons in the VP has also been demonstrated to increase the break point in a PR task (Tooley et al., 2018). The BLA is also known to be involved in valence signaling (Wassum and Izquierdo, 2015), and is thought to contribute to reward-seeking behavior *via* a projection to the NAc (Ambroggi et al., 2008; Stuber et al., 2011). The LH is often referred to as the feeding center of the brain, and activity in an NAc-to-LH pathway has been reported to control consummatory behavior (Maldonado-lrizarry et al., 1995; Stratford and Wirtshafter, 2012; O’Connor et al., 2015). Finally, the DR is a major source of serotonin in the brain regions described above, and the activation of serotonin neurons in the DR has been reported to enhance patience for future rewards (Miyazaki et al., 2014, 2020). Thus, regions of increased centrality in *Kpna3* KO mice have been shown to form neural circuits controlling motivation. Taken altogether, these findings indicate that increased functional connectivity in *Kpna3* KO mice is due to enhanced direct neural connections among the NAc and its upstream/downstream connected brain regions during effortful food-seeking behavior and enhanced this circuit activity may augment motivation in *Kpna3* KO mice. Interestingly, the MPOA was also found to be included within a KO only hub. While the precise role of the MPOA in motivational control is still unknown, it has been reported that this region plays an important role in social reward (McHenry et al., 2017).

In WT mice, in addition to VP and BLA, the orbitofrontal cortex (VO/LO, MO) and median raphe nucleus (MR) also showed high centrality, suggesting that they play important roles in effortful food seeking behavior under normal conditions. It has been suggested that the activity of the orbitofrontal cortex functionally regulates appetitive behavior in the progressive ratio schedule (Cetin et al., 2004; Münster et al., 2020). It should be noted that the OFC also controls motivation *via* its connection to the NAc, but does not increase centrality in KO mice (Hart et al., 2018). Also, it has been reported that the activity of serotonergic projection neurons from the MR to the ventral hippocampus facilitates effortful food-seeking (Yoshida et al., 2019).

In summary, *Kpna3* deficiency augments motivation for reward in a progressive ratio schedule in an operant conditioning task. Human *KPNA3* SNPs have been associated with dependence to drugs of abuse, including ethanol, opiates, and, to a lesser extent, nicotine (Morris et al., 2012). Regardless of the substance, pre-existing personality traits that result in frequent exploratory activities in pursuit of potential rewards are known to be associated with addiction (Hiroi and Agatsuma, 2005). It is possible that KPNA3 depletion may result in a general increase in motivation to seek rewarding stimuli that increases the susceptibility to substance dependence disorders. It is known that the susceptibility to addiction is influenced by genetic factors (Solmi et al., 2021), many of which are regulated by plasticity-related transcription factors including as ΔFosB, CREB, and NFκB family factors (Nestler, 2012). Decreased nuclear localization of such transcription factors due to Importin α depletion may disrupt cell function and lead to changes in motivational behavior. Interestingly, recent studies have revealed that Importin αs participate in synapse-to-nucleus and axon-to-nucleus transport in neurons in addition to their canonical role in nuclear transport (Thompson et al., 2004; Perry and Fainzilber, 2009; Ben-Yaakov et al., 2012; Panayotis et al., 2015). Indeed, Importin αs have been reported to transport CREB2, a transcription factor involved in long-term synaptic plasticity, from the synapse to the nucleus (Lai et al., 2008). Thus, Importin αs may regulate long-term neural responses by transporting transcription factors from the synapse to the nucleus. KPNA3 is expressed in the axons of dorsal root ganglion neurons, and has been confirmed to be involved in retrograde axonal transport by binding to the dynein (Hanz et al., 2003). It is possible that KPNA3 may also regulate neuronal plasticity *via* transport from nerve endings to the nucleus in the hub region identified in this study. Considering the increases in functional connectivity, the loss of synaptic and axonal transport function of KPNA3 may cause increased long-term potentiation or stronger excitatory patterns seen in *Kpna1* KO mice (Panayotis et al., 2018). Further investigation to elucidate the role of KPNA3 in reward-associated brain regions and its relation to functional and dysfunctional reward-related behavior is necessary.

## Data Availability Statement

The original contributions presented in this study are included in the article/Supplementary Material, further inquiries can be directed to the corresponding author/s.

## Ethics Statement

The animal study was reviewed and approved by the Osaka University Institute for Protein Research Animal Committee.

## Author Contributions

YA, KS, TM, TO, YM, YY, MO, and TH contributed to the conception and design of the study and interpretation of data. YA performed the data acquisition and analysis. YA, KS, TM, TO, and TH wrote the manuscript. All authors contributed to manuscript revision, and read and approved the submitted version.

## Conflict of Interest

The authors declare that the research was conducted in the absence of any commercial or financial relationships that could be construed as a potential conflict of interest.

## Publisher’s Note

All claims expressed in this article are solely those of the authors and do not necessarily represent those of their affiliated organizations, or those of the publisher, the editors and the reviewers. Any product that may be evaluated in this article, or claim that may be made by its manufacturer, is not guaranteed or endorsed by the publisher.
