# Supplementary material for: Importin α3 (KPNA3) Deficiency Augments Effortful Reward-Seeking Behavior in Mice
Source: Front Neurosci. 2022 Jun 30;16:905991. doi: 10.3389/fnins.2022.905991 (PMC9279672; doi:10.3389/fnins.2022.905991)
Supplement: Supplementary file 1 [file Image_1.pdf]

Supplementary Material

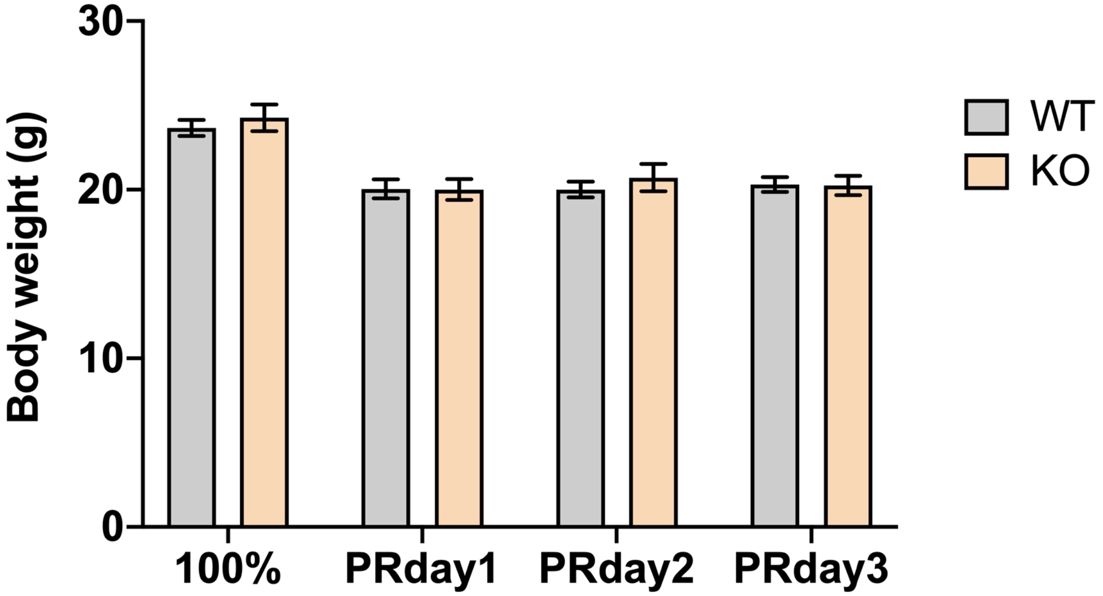

Supplementary Figure 1. Body weight of mice. 100% is body weight before food restriction. PRday1, 2, 3 is body weight recorded just before starting the PR test. There was no significant effect of genotype on body weight (Two-way ANOVA effect of genotype: $F (1, 17) = 0.1518$ $p = 0.7017$; Bonferroni tests between WT and KO: $p > 0.9999$ all days).

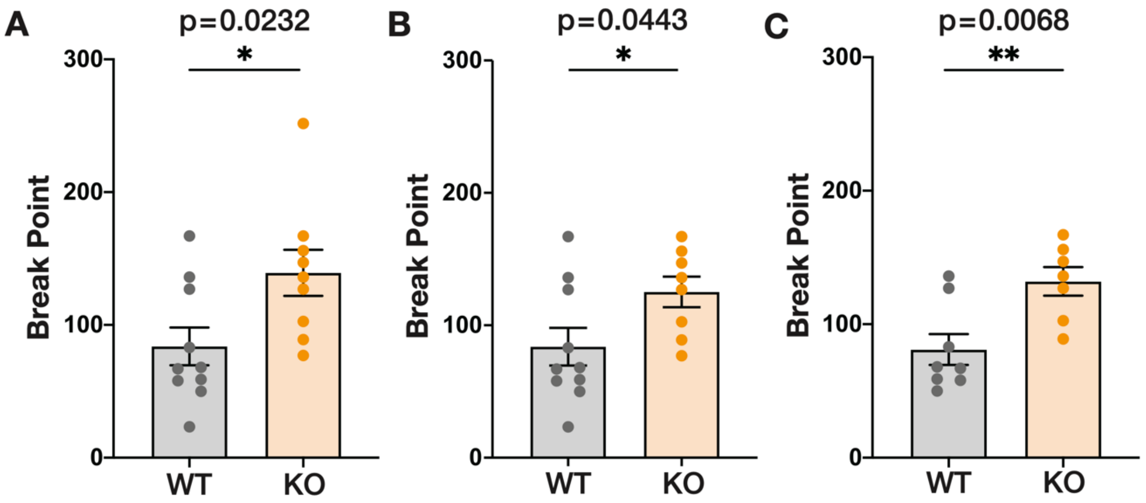

Supplementary Figure 2. Review of outliers**. (A)** Break Point (Same as Figure 2C). The ROUT method (Q:1%) did not detect any outliers in both groups (WT = 10, KO = 9). (B) Data and statistics with the maximum value of the KO group (WT = 10, KO = 8) regarded as an outlier and excluded. (C) Data and statistics with the maximum and minimum values for both groups excluded (WT = 8, KO = 7). Data represent the mean ± SEM, * $p < 0.05$, **: $p<0.01$, Student's t-test.

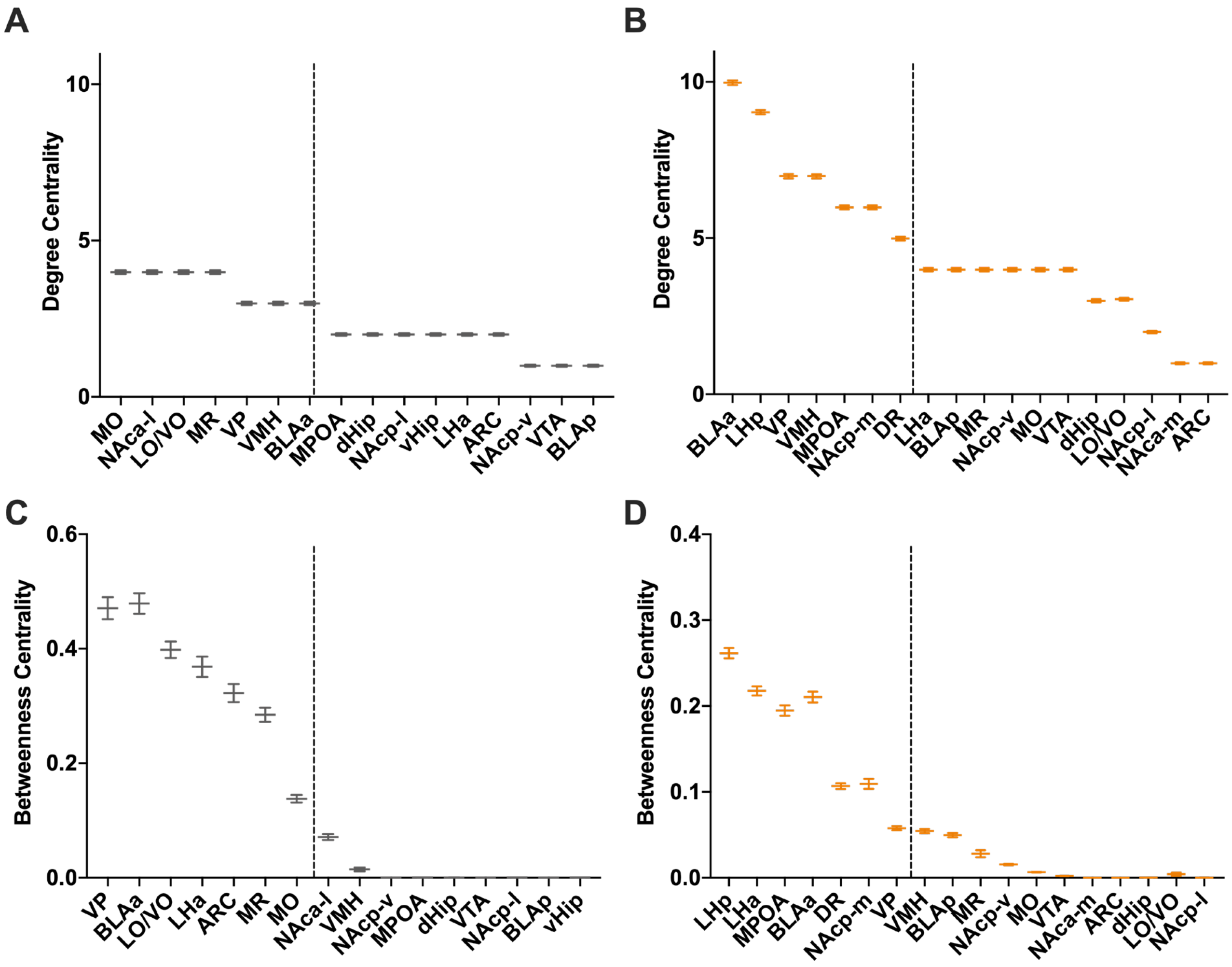

Supplementary Figure 3. Reproducibility evaluation of the centrality score with bootstrap method. (A-D) 95% confidence interval of the centrality score of each region in network. Data represent the mean ± SEM. The order of regions on the horizontal axis is the same as in Figure 5C-D.

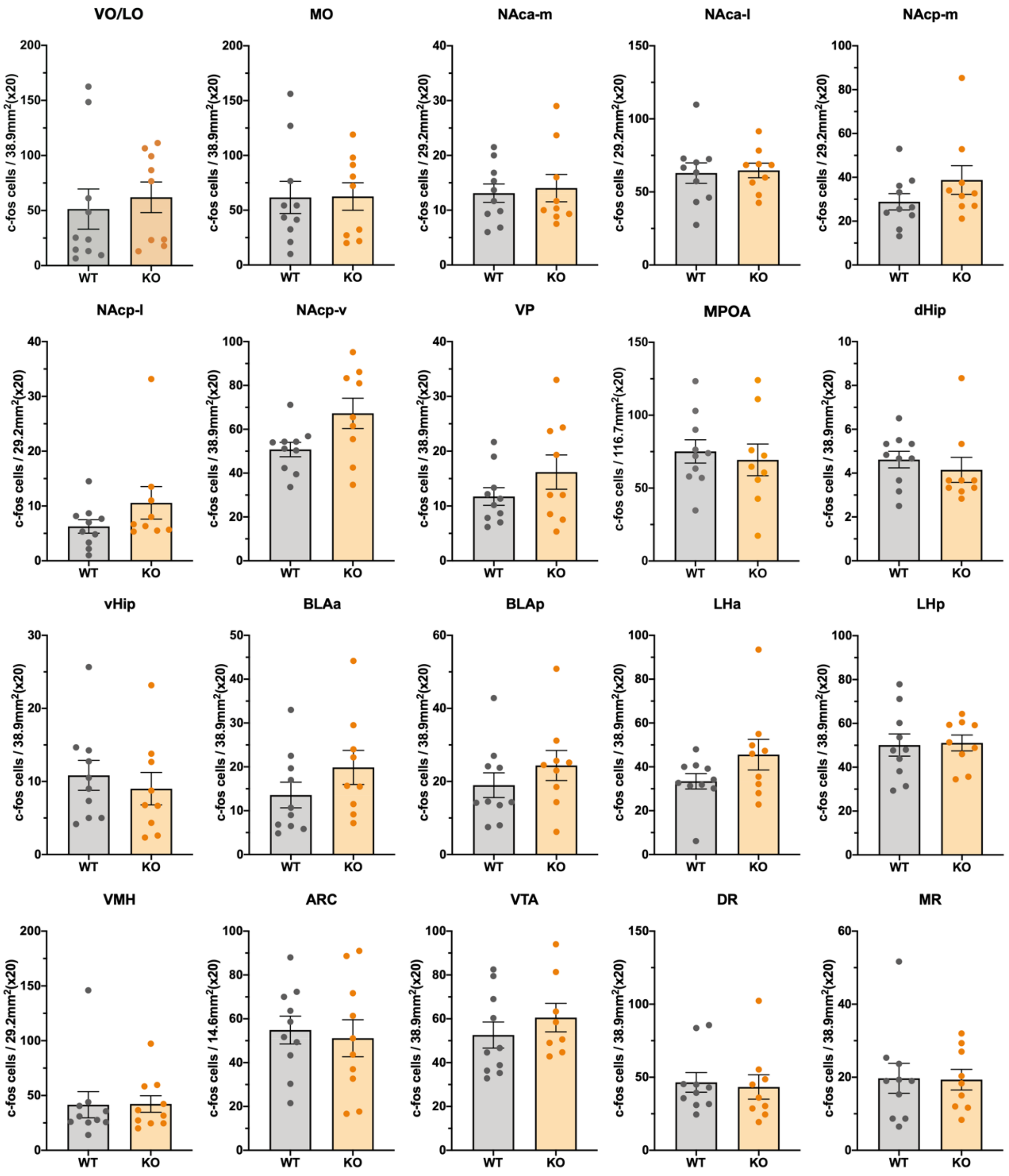

Supplementary Figure 4. The number of c-Fos-positive cells in each region. Data represent the mean ± SEM, a post-hoc Bonferroni multiple comparison test. (WT = 10, KO = 9). ARC: arcuate nucleus, BLA: basolateral amygdala, dHip: dorsal hippocampus, DR: dorsal raphe nucleus, LH: lateral hypothalamus, MO: medial orbital area, MPOA: medial preoptic area, MR: median raphe nucleus, NAc: nucleus accumbens (a: anterior, p: posterior, m: medial, l: lateral, v: ventral), vHip: ventral hippocampus, VMH: ventromedial hypothalamic nucleus, VO/LO: ventral and lateral orbital area, VP: ventral pallidum, VTA: ventral tegmental area

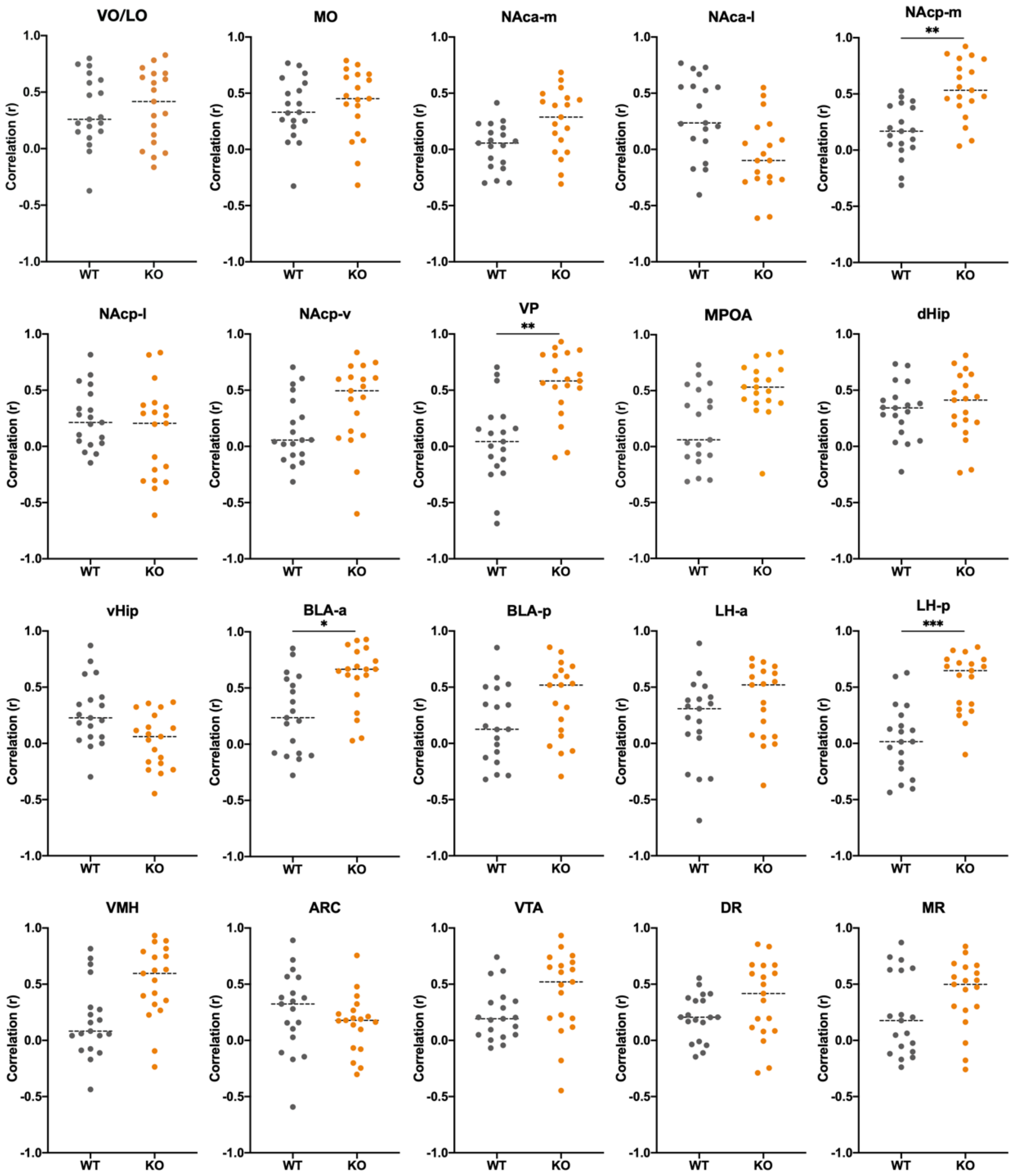

Supplementary Figure 5. Correlation coefficients of the number of c-Fos-positive cells in each region. The solid line indicates the median. Mann Whitney u test and Bonferroni correction, *: p<0.05, **: p<0.01, ***: p<0.0 01. ARC: arcuate nucleus, BLA: basolateral amygdala, dHip: dorsal hippocampus, DR: dorsal raphe nucleus, LH: lateral hypothalamus, MO: medial orbital area, MPOA: medial preoptic area, MR: median raphe nucleus, NAc: nucleus accumbens (a: anterior, p: posterior, m: medial, l: lateral, v: ventral), vHip: ventral hippocampus, VMH: ventromedial hypothalamic nucleus, VO/LO: ventral and lateral orbital area, VP: ventral pallidum, VTA: ventral tegmental area

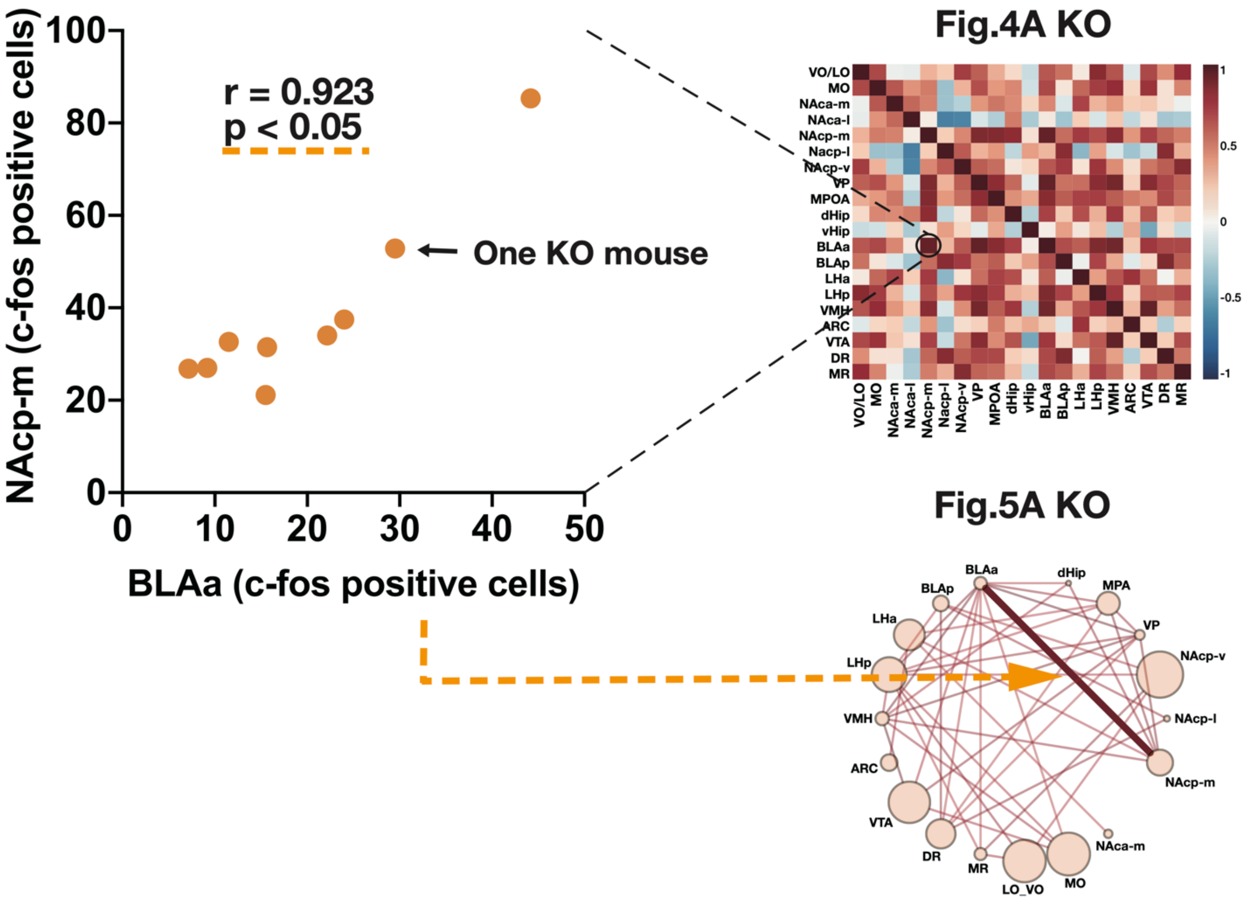

Supplementary Figure 6. Example of correlation coefficient and network construction in KO group
